# Reproducibility of Magnetic Resonance Perfusion Imaging

**DOI:** 10.1371/journal.pone.0089797

**Published:** 2014-02-25

**Authors:** Xiaomeng Zhang, Mark D. Pagel, Amanda F. Baker, Robert J. Gillies

**Affiliations:** 1 Biomedical Engineering Graduate Interdisciplinary Program, University of Arizona, Tucson, Arizona, United States of America; 2 Department of Chemistry and Biochemistry, University of Arizona, Tucson, Arizona, United States of America; 3 University of Arizona Cancer Center, University of Arizona, Tucson, Arizona, United States of America; 4 Hematology/Oncology Section, College of Medicine, University of Arizona, Tucson, Arizona, United States of America; 5 H. Lee Moffitt Cancer Center & Research Institute, Tampa, Florida, United States of America; NIH, United States of America

## Abstract

Dynamic MR biomarkers (T2*-weighted or susceptibility-based and T1-weighted or relaxivity-enhanced) have been applied to assess tumor perfusion and its response to therapies. A significant challenge in the development of reliable biomarkers is a rigorous assessment and optimization of reproducibility. The purpose of this study was to determine the measurement reproducibility of T1-weighted dynamic contrast-enhanced (DCE)-MRI and T2*-weighted dynamic susceptibility contrast (DSC)-MRI with two contrast agents (CA) of different molecular weight (MW): gadopentetate (Gd-DTPA, 0.5 kDa) and Gadomelitol (P792, 6.5 kDa). Each contrast agent was tested with eight mice that had subcutaneous MDA-MB-231 breast xenograft tumors. Each mouse was imaged with a combined DSC-DCE protocol three times within one week to achieve measures of reproducibility. DSC-MRI results were evaluated with a contrast to noise ratio (CNR) efficiency threshold. There was a clear signal drop (>95% probability threshold) in the DSC of normal tissue, while signal changes were minimal or non-existent (<95% probability threshold) in tumors. Mean within-subject coefficient of variation (wCV) of relative blood volume (rBV) in normal tissue was 11.78% for Gd-DTPA and 6.64% for P792. The intra-class correlation coefficient (ICC) of rBV in normal tissue was 0.940 for Gd-DTPA and 0.978 for P792. The inter-subject correlation coefficient was 0.092. Calculated K^trans^ from DCE-MRI showed comparable reproducibility (mean wCV, 5.13% for Gd-DTPA, 8.06% for P792). ICC of K^trans^ showed high intra-subject reproducibility (ICC = 0.999/0.995) and inter-subject heterogeneity (ICC = 0.774). Histograms of K^trans^ distributions for three measurements had high degrees of overlap (sum of difference of the normalized histograms <0.01). These results represent homogeneous intra-subject measurement and heterogeneous inter-subject character of biological population, suggesting that perfusion MRI could be an imaging biomarker to monitor or predict response of disease.

## Introduction

Tumor angiogenesis is a pathophysiological process involving the development of new capillaries and hyperpermeable blood vessels [1]. For a tumor to grow beyond the occult stage (∼1–2 mm diameter), angiogenic activators have to outweigh inhibitors, leading to neovascularization. Activation of the angiogenic process, known as the “angiogenic switch”, is an important step in the progression from small lesions to malignant disease [2]. Thus, it is important to be able to accurately monitor angiogenesis and its response to therapy [3].

### DCE-MRI

Several MRI biomarkers are available to assess the tissue vasculature [4]. MR perfusion techniques using contrast agents (CAs) detect hemodynamic parameters by monitoring the rate of wash-in and wash-out of CAs in the tumor tissue [5]. The common perfusion technique is relaxivity-based DCE-MRI which has been widely used to investigate angiogenesis within tumors, and in particular the response to antiangiogenic therapy. The longest experience is with Gd-diethylenetriamine pentaacetic acid (Gd-DTPA), which has been used since the 1980s. Kinetic models have been applied in order to derive estimates of tissue perfusion and permeability based on the slope of the tumor wash-in and/or wash-out curves [5], [6]. Many applications of quantitative DCE-MRI to detect response to anti-angiogenic and anti-vascular drug treatments assume that these methods are reproducible and can be used to predict biological changes [7]. The inherent value of a biomarker is related to its biological variability relative to the reliability of measurement. Test-retest reproducibility of these biomarkers are important but have rarely been estimated [8]. The current study evaluated the reproducibility of DCE-MRI with two contrast agents that have different molecular weights and hydrodynamic radii. In addition, we hypothesized that the larger CA, P792, is more sensitive to permeability over blood flow, but has a lower contrast-to-noise ratio (CNR) due to slow extravasation.

### DSC-MRI

An Anti-angiogenic therapy, such as Avastin (bevacizumab) that targets vascular endothelial growth factor, prevents the growth of new vessels and induce regression of immature blood vessels. Paradoxically, instead of reducing perfusion, these therapies can result in the “normalization” of vessels and improved perfusion [9]. This is important as it can increase the efficacy of subsequent radiation or drug delivery, resulting in better response. Thus, combination therapies with an anti-angiogenic agent followed by a targeted or cytotoxic agent are becoming an important anti-cancer paradigm [10]. DSC-MRI is a rapid imaging technique that can yield quantitative estimates of microvasculature changes, which may aid in identifying the normalization time window for pre-clinical chemotherapy studies [11]. This relies on compartmentalization of the contrast agent while a susceptibility difference can be induced between intravascular and extravascular spaces. Signal loss caused by spin dephasing during the first pass of CA circulation is associated with the magnetic field distortions in the vicinity of vessels. The susceptibility differences could also be affected by vascular permeability and tortuosity. Therefore, time-course T2* signal change could be an indicator of vascular permeability and tortuosity in tumors.

### Contrast agents

MR contrast agents that are marginally permeable to normal vascular endothelium are able to extravasate more rapidly through the endothelium of angiogenic tumor vessels to produce differential image enhancement. This results in a fast wash-in of CA coupled with a moderate to rapid wash-out and allows for a functional analysis of the tumor vascular permeability. However, the kinetic parameter K^trans^ measured with MRI contrast agents is dependent on both blood flow and permeability [6]. When blood flow is low relative to permeability, the amount of CA that moves into the extracellular extravascular space will depend primarily on the supplied rate to the endothelium, and K^trans^ will reflect blood flow in a “flow-limited” state. If CA delivery is not limited by blood flow relative to permeability, K^trans^ reflects permeability in a “permeability-limited” state. Thus, high values of K^trans^ indicate high permeability and probably high blood flow, while low values of K^trans^ indicate low permeability and/or low blood flow. In either case, change in K^trans^ following therapy likely represents a genuine pharmacodynamic effect of the therapy if subject physiology is consistently maintained during the MRI scan session (e.g., core body temperature and level of anesthesia are carefully regulated).

In theory and practice, the pharmacokinetic rates measured with MRI are affected by the hydrodynamic radius of the CA [12]. Gd-DTPA is a commonly used CA with a hydrodynamic radius of 0.9 nm. This small molecule CA is a freely diffusible tracer and yields a K^trans^ that is more proportional to the blood flow. P792 is a monogadolinium chelate based on a cyclen structure with semi-rigid hydrophilic ligands, which has a hydrodynamic radius of ca. 6 nm that is similar to that of a macromolecular CA [13]. P792 is potentially more suitable for selective imaging of the tumor neovasculature that tends to be more permeable to macromolecules than normal tissues. P792 is a minimally diffusible tracer and yields a K^trans^ that may more accurately reflect permeability within tumors. For these reasons, macromolecular agents are being increasingly investigated because of these inherent advantages [14]. However, to be useful as a biomarker, especially to measure therapy-induced changes in vascular permeability, the reproducibility of kinetic parameters must be assessed. In this paper, we compare the reproducibility of small and large molecular weight CAs using a standard acquisition protocol and quantitative analysis method in a mouse tumor model.

To measure an early response to cancer therapy during pre-clinical studies, a threshold in vascular biomarkers between drug-treated and vehicle cohorts needs be determined. In clinical studies, wCV values for K^trans^ have been found to be in the range of 24% to 29% in various tumors, and changes of 45% to 83% in perfusion measurements have been needed to overcome the variability of measurements [15]. A suggested change of 50% has been widely chosen based on another group's repeatability data [16]. These examples suggest that it is difficult to justify the use of imaging biomarkers in vivo unless there is a good understanding of reproducibility.

## Materials and Methods

### Cell lines and animals model

MDA-MB-231 breast cancer cells were obtained from the American Type Culture Collection (ATCC) and grown in Dulbecco's Modified Eagle Media supplemented with 10% fetal calf serum and antibiotics in a 5% CO_2_ humidified incubator at 37°C. Cells were routinely monitored for mycoplama contamination and cell line authenticity. Orthotopic tumors were obtained by injecting female SCID mice with 10×10^6^ cells into the mammary fat pad (MFP). Tumors were allowed to grow for three weeks and measured using electronic calipers and tumor volumes calculated as π/6[(short axis in mm)^2^×(long axis in mm)]. The animals were sacrificed when tumors reached 2000 mm^3^. Animal protocols were approved by the University of Arizona Institutional Animal Care and Use Committee.

### Magnetic resonance imaging

A total of sixteen mice underwent MRI studies. Each contrast agent was tested with eight mice that had subcutaneous MDA-MB-231 breast xenograft tumors. Each mouse was imaged with a combined DSC-DCE protocol three times within one week to achieve measures of reproducibility. Each animal has 48 hours to recover before next scan to ensure adequate time for contrast agent washout and physiologic recovery.

MRI experiments were performed with a Bruker Biospec 7 T MRI scanner equipped with a maximum gradient amplitude of 600 mT/m. All animals were anesthetized by inhaled isoflurane (1.5% in O_2_) at 1.0 liters per minute and cannulated at the tail vein. A pressure-transducer pad was taped to the animal's chest to continuously monitor its respiration rate. This respiratory rate was monitored at a range of 60-90 breaths per minute during the course of the imaging experiments. Body temperatures were continuously monitored using a rectal fluoroptic thermometer (SAII®, SA Instruments, Stony Brook, NY). An external heater was used to maintain body temperature at 37.0±0.2°C. The animal was gently secured in a plastic holder and loaded into a small animal imaging Litz coil with a 34 mm inner diameter (Doty Scientific, Columbia, SC, USA). *T*_2_-weighted images were acquired using a rapid acquisition with relaxation enhancement (RARE) MRI pulse sequence with a RARE factor of 8, giving an effective time of evolution (TE) of 72 ms. A series of spin echo (SE) images at six different time of recovery (TR) values were acquired prior to injection of the contrast agent (TR = 200, 400, 800, 1500, 3000, 5000 ms, TE = 8.5 ms, field of view (FOV) = 35×35 mm, matrix = 128×128, spatial resolution = 273×273 µm). A 1.5-mm-thick axial slice was oriented through the center of the tumor and three additional slices were oriented through the thighs to monitor the contrast agent in the femoral artery to obtain a vascular input function (VIF). The thighs were tightly secured with tape to avoid motion artifacts. During bolus administration of a single dose of CA (0.1 mmol/kg for P792 or 0.25 mmol/kg for Gd-DTPA injected within 5 s), a GRadient-Echo Fast Low Angle SHot (GRE FLASH) MRI pulse sequence was applied for 60 seconds (DSC-MRI, TR = 10 ms, TE = 5 ms, flip angle = 5°, slice thickness = 1.5 mm, FOV = 35×35 mm, matrix = 64×64, spatial resolution = 547×547 µm, temporal resolution = 0.6 s) followed by a dynamic series of spin echo T1-weighted images (DCE-MRI, TR = 150 ms, TE = 7.2 ms, Slice = 1.5 mm, FOV = 35×35 mm, matrix = 128×128, spatial resolution = 273×273 µm, steady state scans = 2) for 30 minutes. The reduced spatial resolution of DSC images was compromised to maximize the temporal resolution for making perfusion measurements. A 5° flip angle was used for the GRE FLASH sequence to minimize the T1 relaxation effect on DSC-MRI results.

### T2* DSC-MRI Analysis

**Figure 1:**
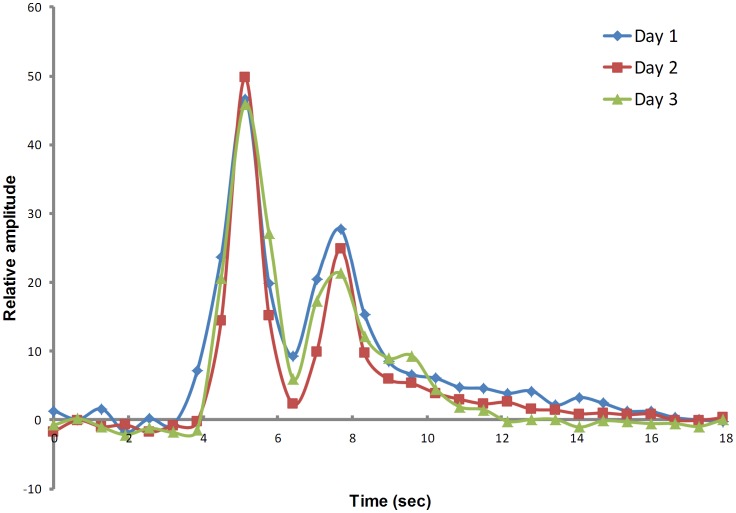
ΔR2* calculated from normal tissue in a single mouse acquired on three different days. In this case, first- and second- pass circulation of CA injection is observed. Only first –pass circulation was used in DSC analysis.



The concentration of a CA is directly proportional to a change in relaxation rate (ΔR2*) [17], which can be calculated for each time point of the T2*-weighted DSC-MRI images (Figure 1):

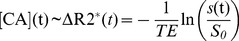
(1)where [CA](t) is contrast agent concentration in blood at time t, S_0_ and S(t) are the signal amplitudes at the baseline prior to injection and at time t after injection, respectively, and TE is the echo time of the MR sequence used. The relative blood volume (rBV) (arbitrary units) is then the integral of the R_2_*-time curve:



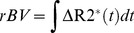
(2)


The relative mean transit time (rMTT, in units of seconds) was approximated by measuring the width of the ΔR2*-time curve at half its maximum value (full-width, half-maximum). Then relative blood flow (rBF) was obtained by substituting the transit time equation of the central volume theorem [18]:



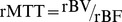
(3)


A single global value for the entire ROI was obtained from the mean of voxel values.

The CNR was used to quantitatively distinguish the signal changes from baseline noise of DSC data. A CNR of 2√2 multiplied by the standard deviation of the normalized baseline value represents >95% probability that the signal change was not due to random noise fluctuations, assuming that the signal to noise ratio (SNR) is high so that noise can be approximated to be a Gaussian distribution [19].

### Pharmacokinetic Modeling of DCE-MRI

Although algorithms have been developed to assess the initial uptake of a MRI contrast agent into the tumor tissue, these methods require rapid temporal sampling rates and bolus injections to achieve accurate results. An alternative algorithm can assess DCE-MRI results after the initial uptake has reached steady-state, which is less sensitive to the temporal sampling rate and to having a bolus injection, and can be easily and rapidly evaluated by using the Patlak graphical method [20]. This pharmacokinetic model assumes that contrast agent leaks at a uniform rate from the blood pool into tissue extra-cellular space but does not leak back into the blood pool, and furthermore it critically depends on the VIF to account for different injection amounts and durations of each protocol, as well as different physiological elimination rates during each DCE MRI scan session [21]. Classically, a VIF can be measured with MRI by directly visualizing an artery or vein in the MR image.

### DCE-MRI Analysis

K^trans^ values can be calculated by simplified equation derived from the Kety models and the Patlak method (Eq. (4)). A complete description of this method can be found in references [22] and [23].



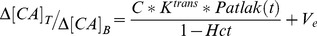
(4)where,

Δ [CA]_T_: Change in CA concentration in tumor

Δ [CA]_B_: Change in CA concentration in blood pool

c: Constant dependent on MRI acquisition parameters

K^trans^: Permeability-surface area volume transfer constant

Patlak(t): Patlak time, accounts for blood CA clearance

Hct: Hematocrit, fraction of red blood cells in blood volume

V_e_: Plasma volume of extracellular & vascular space

### Data and statistical analysis

MRI data were analyzed using self-developed programs written for MATLAB (Mathworks, Natick, MA). A region of interest (ROI) encompassing the tumor was drawn manually on the T2-W anatomical image. The ROIs for obtaining the VIF were determined by a self-constructed automatic selection algorithm. This algorithm first segmented all possible artery/vein ROIs by comparing the signal amplitude differences of pre- and post-CA, then calculates the slopes of time-course signal curve for these ROIs and sorts slopes within a reasonable range into a candidate pool, and finally averages the most similar slopes into a VIF. A pixel-wise temporal smoothing method was performed with a 6-degree spline interpolation. A Gaussian spatial smoothing was then formed with a 3×3 matrix with center to edge ratio of 9. A map of K^trans^ values within the ROI were used to generate normalized distribution histograms and cumulative histograms. Cumulative histograms were constructed by counting the cumulative number of observations in normalized histograms. The Area Under the Curve (AUC) was calculated from each histogram. The differences of AUC for three repeated measurement of a single animal were used to quantify the reproducibility. A diference approaching 0 indicates higher reproducibility. Statistics of reproducibility between three scans per mouse were determined using wCV (Eq. (5)) and ICC (Eq. (6)).



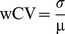
(5)


Where σ is standard deviation and μ is mean value.



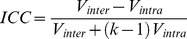
(6)where V_inter_  =  inter-subject variance and V_intra_  =  intra-insubject variance. This ICC captures the differences between inter and intra-subject variability [24]. For instance, if intra-subject variability is very low, indicating good intra-subject reliability, then the ICC is close to one. For the analysis of ICC, the Statistical Package for Social Sciences Windows (SPSS Inc., Chicago, Illinois, USA) was used. Intra-class ICC was calculated from a group of animals using the same CA, while inter-class ICC was calculated from all animals between two different CA groups.

## Results

DCE-MRI results were also obtained for both contrast agents in all animals. For K^trans^ calculated from DCE-MRI, the mean wCV was 5.13% for the Gd-DTPA group and 8.06% for the P792 group. The ICC for intra-subject reproducibility was 0.999 for the Gd-DTPA group and 0.995 for the P792 group. The inter-subject reproducibility measured by ICC was 0.774 (Table 1). One animal died during the first scan and a second animal died during the third scan, and two additional image sets had significant motion artifacts at the tumor location. These sets of images were excluded from subsequent analyses.

**Table 1:** Summary of the K^trans^ reproducibility measurements.

Animal Index/CA	Number of Scans	Mean (min^−1^)	STD (min^−1^)	wCV (%)	Mean wCV (%)	ICC for intra-subject reproducibility	ICC for inter-subject reproducibility
**01/Gd-DTPA**	3	0.46	0.02	3.31	5.13	0.999	0.774
**02/Gd-DTPA**	3	0.13	0.00	1.68			
**03/Gd-DTPA**	1	-	-	-			
**04/Gd-DTPA**	2	0.82	0.01	1.27			
**05/Gd-DTPA**	3	0.30	0.03	9.89			
**06/Gd-DTPA**	3	0.38	0.02	5.27			
**07/Gd-DTPA**	3	0.15	0.01	4.18			
**08/Gd-DTPA**	3	0.26	0.03	10.32			
**09/P792**	3	0.47	0.01	3.06	8.06	0.995	
**10/P792**	3	0.27	0.02	7.09			
**11/P792**	3	0.26	0.02	7.65			
**12/P792**	3	0.13	0.01	8.53			
**13/P792**	2	0.98	0.02	2.11			
**14/P792**	3	0.13	0.02	14.76			
**15/P792**	3	0.10	0.02	16.85			
**16/P792**	2	0.63	0.03	4.46			

**Figure 2:**
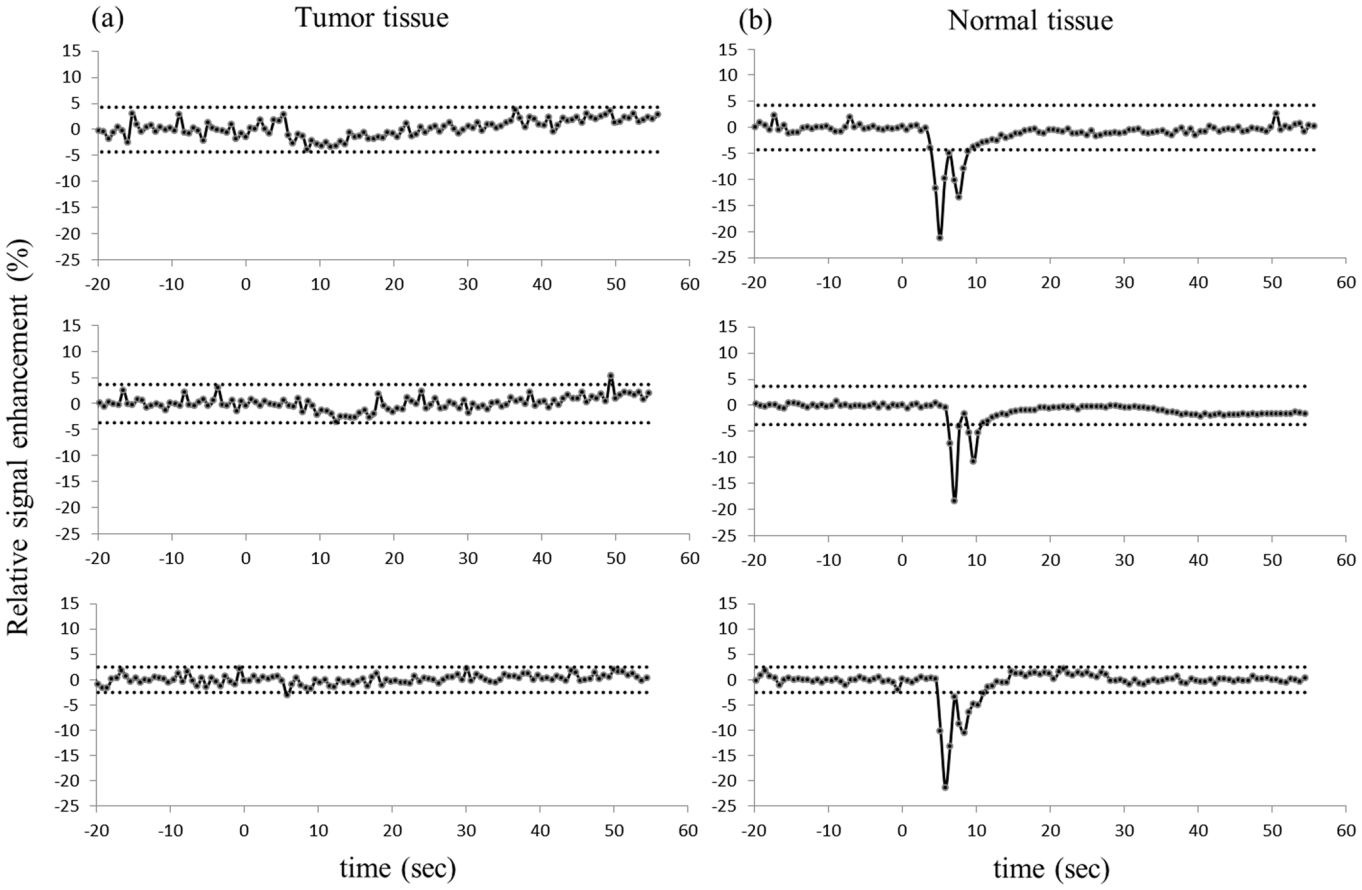
Signal amplitude change of DSC on tumor tissue (left column) and normal tissue (right column). Three rows represent three separate measurements (top row: Day 1; middle row: Day 3; bottom row: Day 5) for a single animal. The 95% confidence limits for significant changes in signal are shown on each graph (dot line), The bolus was injected at 0 seconds.

DSC imaging data were also measured between all animals with both agents. In all cases, the DSC results were able to distinguish the tissue vasculature and tumor. Three repeated exams of each single animal showed a statistically significant drop in R2* signal in normal tissue (threshold at 95% confidence interval) (Figure 2b), while R2* signal changes did not exceed the threshold for statistical significance within tumor tissue (Figure 2a). The values of rBV, rBF and MTT were also estimated (Eq. (1-3)). For the rBV calculated from DSC-MRI results, the mean wCV was 11.78% for the Gd-DTPA group and 6.64% for the P792 group. The ICC for intra-subject reproducibility was 0.940 for the Gd-DTPA group and 0.978 for the P792 group. The inter-subject reproducibility measured by ICC was 0.092 (Table 2).

**Table 2:** Summary of the rBV reproducibility measurements. Arbitrary units (AU).

Animal Index/CA	Number of Scans	Mean (AU)	std (AU)	wCV (%)	Mean wCV (%)	ICC for intra-subject reproducibility	ICC for inter-subject reproducibility
**01/Gd-DTPA**	3	43.18	2.58	5.97	11.78	0.94	0.092
**02/Gd-DTPA**	3	93.27	3.27	3.51			
**03/Gd-DTPA**	1	-	-	-			
**04/Gd-DTPA**	2	73.85	3.89	5.27			
**05/Gd-DTPA**	3	50.42	4.01	7.95			
**06/Gd-DTPA**	3	38.04	7.37	19.38			
**07/Gd-DTPA**	3	71.23	16.47	23.12			
**08/Gd-DTPA**	3	34.15	5.90	17.26			
**09/P792**	3	47.56	2.19	4.61	6.64	0.978	
**10/P792**	3	63.36	1.67	2.64			
**11/P792**	3	69.61	4.68	6.72			
**12/P792**	3	63.05	1.63	2.58			
**13/P792**	2	55.46	2.25	4.05			
**14/P792**	3	96.90	10.58	10.91			
**15/P792**	3	46.82	6.15	13.13			
**16/P792**	2	136.68	11.54	8.44			

**Figure 3:**
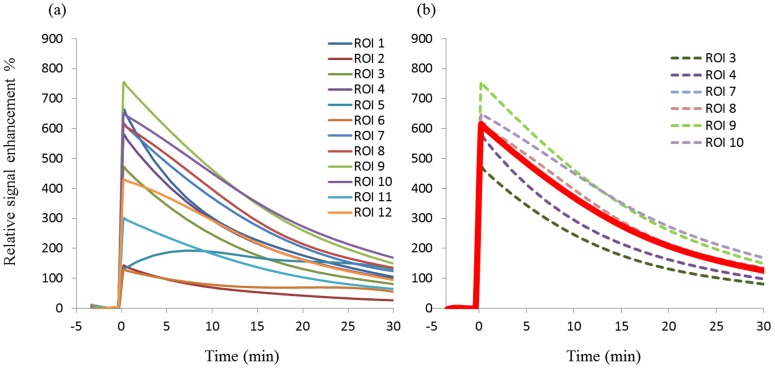
VIF auto-selection algorithm. VIFs are selected by comparing the signal change between pre- and post- injection (a); VIFs were further restrained (dot line) by comparing their slopes (b); The final VIF was the average of VIFs with similar slopes (thick red line).

**Figure 4:**
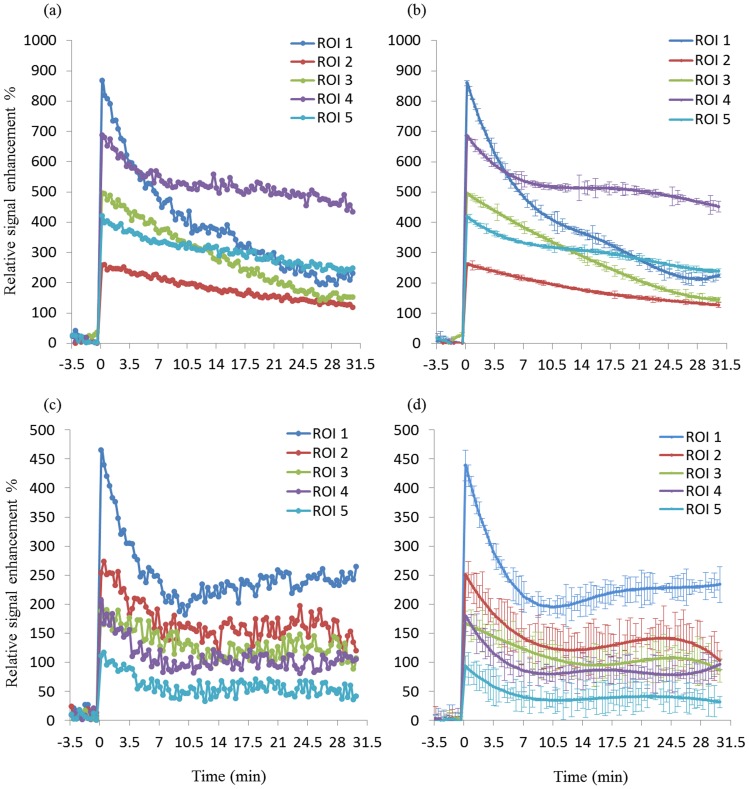
VIFs from Gd-DTPA (a) and P792 (c). VIFs were smoothed by temporal spline-fit function for Gd-DTPA (b) and P792 (d).

**Figure 5:**
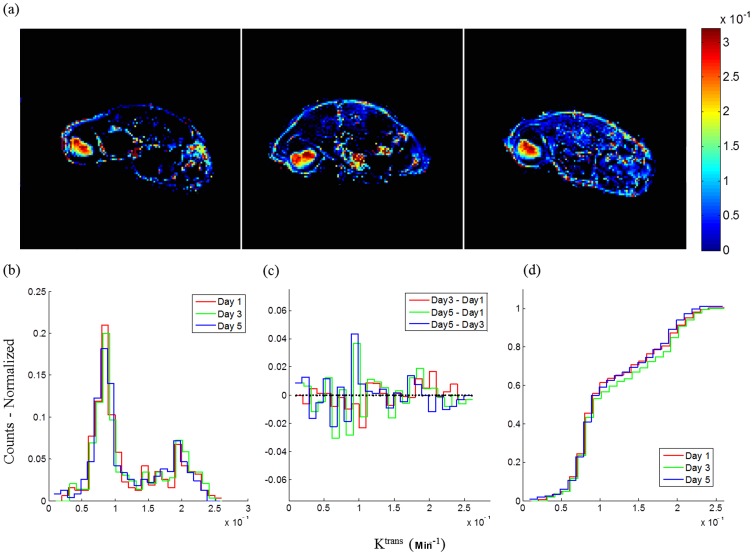
K^trans^ parametric maps of a single mouse acquired on three different days (Day 1, Day 3 and Day 5) within one week (a); Histogram of K^trans^ in tumor ROI for all 3 days (b); Difference of histograms (c). Cumulative histograms of K^trans^ in tumor ROI on three different imaging days (d).

The kinetic parameters of DCE-MRI were estimated on a pixel wise basis by applying the Kety model and Patlak method. Calculated values were observed to be highly dependent on the proper determination of the unique VIF in individual subjects, suggesting that this pharmacokinetic modeling is sensitive to the measurement of VIF. However, the ROI selection for artery or vein is difficult especially in a small animals due to the size of the vessels [21]. The VIFs in this study were measured with a multi-ROI selection procedure. As an example, twelve candidate ROIs were selected by the auto-selection algorithm in one animal (Figure 3a). These ROIs were further restrained by comparing time-course signal curve slopes (Figure 3b). The slopes that were within a 10% variability were averaged, which generated a more reliable VIF slope compared to a manually selected single ROI that putatively represented the VIF, and thus yielding more reproducible kinetic values for each individual mouse. To compensate for the lower image SNR produced by P792 relative to Gd-DTPA, a temporal spline-fit of the VIF reduced the effect of noise in the VIF from subsequent analysis steps (Figure 4c–d), resulting in comparable permeability maps to Gd-DTPA. Motion artifacts are another important impact factor on reproducibility. For tumor image slices, a centrally weighed Gaussian smoothing kernel was applied to reduce these errors. No significant motion occurred in thigh image slices as a result of firmly taping the thighs to the cradle. The kinetic parameter K^trans^ was then calculated pixel-wise for each animal at different scan days and these data were used to generate a mean value. Analyses of these data showed high inter-subject variability of mean K^trans^ values between animals from 9.9×10^−5^ to 1.28×10^−3^, but no significant intra-subject differences were observed in any individual animal. The ICCs for K^trans^ with associated 95% confidence limits confirmed this conclusion (Table 1). This two-way mixed correlation coefficient model illustrates the expected reliability of repeated measures derived from groups of animals where the expected direction of change is unknown. Distributed histogram analyses from three scans of all animals were used to visualize the reproducibility of the DCE-MRI results. The K^trans^ distribution histograms for three measurements of each mouse had a high degree of overlap and the sum of the AUC value generated by the difference of the histograms was small (−0.0066), which indicated high reproducibility (Figure 5c). Furthermore, cumulative histograms were calculated and consistent with the distribution histograms (Figure 5d). These data show high intra-subject reproducibility (i.e., different measurements of single subject) and high inter-subject biological variability. These results showed that there is no significant difference in the measured K^trans^ values between the two CAs.

## Discussion

Although a vast number of studies have used this technique, the issue of reproducibility has often been underappreciated Only a few studies have addressed the reproducibility of DCE-MRI [15], [25]. Reproducibility is especially important for the application of assessment of tumor response to treatment [26]. Additionally, there are few studies comparing the reproducibility of different MW contrast agents.

This study demonstrated the reproducibility of DCE-MRI with CAs that have small and large molecular weights, and using standard MRI acquisition and analysis protocols in a breast xenograft tumor model. A major finding in this work was that the VIF was highly variable and needs to be calculated for each experimental setup. An automated VIF ROI selection method was developed that averaged time activity from voxels that were identified using pre-defined kinetic thresholds. Using these values, a low wCV of K^trans^ were obtained. This indicates that this measure of tumor vascular permeability can be measured with excellent reproducibility, and has higher ICC value than previously measured [27]. Results of earlier studies have shown a range of wCV between 6% and 29% for K^trans^ [15], [25], [28]. Evaluating the intra-subject ICC was required because only evaluating wCV is insufficient to assess reproducibility, as there are some limitations to the use of wCV for agreement between techniques [29]. The low inter-subject ICC value for K^trans^ (0.774) illustrates the wide range of variation, which represents high biological variability among tumors.

Although reproducibility is a widely accepted term, in practice most MRI studies do not directly reproduce imaging results from the same tissue position, especially during longitudinal studies. Previous studies often reported failures on pixel-wise test-retest analyses with a resultant pixel exclusion ranging from 13 to 74% [15], [28]. Instead, the distribution of K^trans^ values across all pixels within the tumor ROI is a more robust comparison between image sets. A histogram can be used to graphically summarize and display the distribution of K^trans^ (Figure 5b). Temporal changes in parameters can be visualized with the use of histograms that show the distribution of parameter values measured throughout the tumor. Such distribution analysis is sensitive to tumor progression and response to therapy. These distributions of K^trans^ in tumors from most animals measured in three different scans were overlaid and were observed to be essentially identical. The difference of AUCs from histograms, which provided more visual information about variation within a tumor, further demonstrated high reproducibility of results from individual tumors.

The detection of a reproducible VIF is crucial to obtain reproducible estimates of kinetic parameters. Multiple factors for VIF estimation that may induce errors include partial volume effects, low temporal and spatial resolution, low contrast to noise ratio (CNR) and in many DCE-MRI cases, a poor choice of major arteries in the field of view and motion artifacts. In our study, the VIF was estimated using the contrast agent enhancement data from three T1-weighted images of the thigh. Multiple ROIs were selected using an automatic detection algorithm that traces the signal changes of each pixel through all slices. Potential problems with motion artifacts and low SNR were mitigated by using a temporal spline-fit function and a spatial Gaussian kernel to smooth the data for pixel-wise analyses. The algorithm-selected ROIs gave a more reproducible VIF compared to manual selection and thus produced more reliable K^trans^ values.

In this study, we used Gd-DTPA and CA P792 that represent a small molecular weight and a large molecular weight CA, respectively. Despite a higher molar relaxivity and comparable injected concentrations, the larger size of P792 limited the amount of CA that perfused through the endothelial wall, which produced lower CNR resulting in lower reproducibility relative to measurements with the small molecule Gd(III) chelate. Data smoothing could compensate for low CNR and generate comparable results. We had hypothesized that the larger CA would be more reproducible, as it is more sensitive to permeability and less sensitive to flow effects. However, this was not show by our study, which did not discriminate the CAs on the basis of their reproducibility, and instead showed similar reproducibilities.

Our results indicated T2* effects associated with the first pass of CAs, which occurred approximately 3–5 seconds after bolus injection, were detectable in normal tissue but not in tumor tissue. The minimal change of DSC-MRI signal in tumors can be ascribed to the decreased tumor blood flow due to the ‘steal’ effect [30], which reduces R2* by decreasing the concentration of the CA into the tumor in the first-pass of circulation. This lack of signal change in tumors may also be due to the incoherent organization of the tumor vasculature. An incoherent vasculature could reduce susceptibility effects, as they would be averaged out over all orientations [31]. For comparison, T1-weighted DCE-MRI of the same tumors showed a time-dependent increase in signal intensity, ascribed to extravasation.

One limitation of this in-vivo reproducibility study is the assumption that the tumor vasculature maintains the same characteristics during the test-retest time window. Although tumor volumes experienced only a minor change in this study (wCV = 2.05%), it is possible that some tumors had significant changes in its vasculature during the scan interval. However, a 48 hour recovery time between tests for each animal was necessary for contrast agent washout and physiologic recovery e.g. from the hypophagia of anaesthesia.

## Conclusion

Intra-subject reproducibility tests for perfusion MRI are a fundamental requirement for many biomarker studies. Inter-subject variability presents a blueprint of biological heterogeneity and complexity. We conclude that tumor vascularity characteristics can be measured reproducibly using DCE-MRI in the same mouse when imaging analysis is carefully performed. Reproducibility of DCE-MRI results may lend confidence in measuring or predicting the effect of anti-angiogenic therapies. DSC-MRI consistently detected the first pass of each contrast agent in normal tissue, but did not detect the first passes in tumor tissues, suggesting that this technique has potential to assess abnormal tumor vessels.
